# A qualitative study of the experience and impact of neuropathic pain in people living with HIV

**DOI:** 10.1097/j.pain.0000000000001783

**Published:** 2020-12-20

**Authors:** Whitney Scott, Maite Garcia Calderon Mendoza del Solar, Harriet Kemp, Lance M. McCracken, Amanda C de C Williams, Andrew S.C. Rice

**Affiliations:** a Health Psychology Section, Institute of Psychiatry, Psychology, and Neuroscience, King's College London, London, United Kingdom; b INPUT Pain Management Unit, Guy's and St Thomas' NHS Foundation Trust, London, United Kingdom; c Department of Surgery and Cancer, Pain Research Group, Faculty of Medicine, Imperial College London, London, United Kingdom; d Department of Psychology, Uppsala University, Uppsala, Sweden; e Research Department of Clinical, Educational, and Health Psychology, University College London, London, United Kingdom

**Keywords:** HIV, Neuropathic pain, Psychosocial, Qualitative

## Abstract

Painful distal symmetrical polyneuropathy is common in HIV and is associated with reduced quality of life. Research has not explored the experience of neuropathic pain in people with HIV from a person-centred perspective. Therefore, a qualitative interview study was conducted to more deeply understand the experience and impact of neuropathic pain in this population. Semistructured interviews were conducted with 26 people with HIV and peripheral neuropathic pain symptoms. Interviews explored the impact of pain and participants' pain management strategies. Interviews were transcribed verbatim and analysed using thematic analysis. Four themes and 11 subthemes were identified. Theme 1 reflects the complex characterisation of neuropathic pain, including the perceived unusual nature of this pain and diagnostic uncertainty. Theme 2 centred on the interconnected impacts of pain on mood and functioning and includes how pain disrupts relationships and threatens social inclusion. Theme 3 reflects the struggle for pain relief, including participants' attempts to “exhaust all options” and limited success in finding lasting relief. The final theme describes how pain management is complicated by living with HIV; this theme includes the influence of HIV stigma on pain communication and pain as an unwanted reminder of HIV. These data support the relevance of investigating and targeting psychosocial factors to manage neuropathic pain in HIV.

## Abstract 2

Supplemental Digital Content is Available in the Text.

These qualitative data support the relevance of investigating and targeting psychosocial factors to manage neuropathic pain in people with HIV.

## 1. Introduction

Combined antiretroviral therapy (cART) has dramatically changed the clinical management of HIV.^36^ As people with HIV in some parts of the world have near normal life expectancy,^52,67^ there is growing emphasis on quality of life. Poorly managed pain is a predominant threat to quality of life in the current cART era.^43^ Pain of any aetiology is reported by 54% to 83% of people with HIV.^43,51^ Painful distal symmetrical polyneuropathy is characterised by axonal degeneration and may be a complication of the virus or neurotoxic antiretroviral drugs, such as the nucleoside reverse transcriptase inhibitors (eg, stavudine).^29^ HIV-related neuropathic pain in the current era affects approximately 22% to 44% of people with HIV,^18,48,72^ compared with a neuropathic pain prevalence of 7% to 8% in the general population.^5,64^ Neuropathic pain in HIV is strongly associated with reduced quality of life.^18^

Pharmacological treatments for HIV-related neuropathic pain have limited effectiveness.^12,15,46,59^ Moreover, analgesics that are helpful for neuropathic pain in other conditions (eg, pregabalin in diabetic neuropathy) are not effective for HIV-related neuropathic pain.^21,46^ Beyond medical management, there is good evidence that psychological interventions, such as cognitive-behavioural therapy (CBT), improve quality of life for people with chronic pain.^75^ However, limited research has investigated CBT for pain in HIV, and studies have had high drop-outs^19^ and small samples.^66^

Improving psychological treatments for chronic pain in HIV will require a better understanding of the psychosocial complexities associated with pain in this population. A recent systematic review of 46 quantitative studies found evidence for associations between HIV-related pain and depression, post-traumatic stress, drug abuse, sleep disturbance, and unemployment.^55^ Persistent pain is also associated with missed HIV clinic visits and reduced ART adherence^55^; the latter is crucially important given the association between ART nonadherence and morbidity and mortality.^63^ Most of the studies in this review reported on samples of mixed or unspecified pain aetiology, predominantly from the United States.^55^ Therefore, the applicability of these findings to neuropathic pain and to other contexts is uncertain.

Building on the quantitative literature, Merlin et al.^40^ conducted a qualitative study of pain in HIV in a predominantly male, African American sample in Alabama, USA. Key themes included bidirectional relationships between pain and low mood, self-medication with illicit drugs, and difficulties accessing prescribed analgesics.^40^ This study did not specify the aetiology of participants' pain nor did it explore how pain type (eg, neuropathic pain) might impact on factors such as low mood. Further exploration of the identified themes in other populations most commonly affected by HIV, namely men who have sex with men, women, and people from diverse racial/ethnic backgrounds, is needed.^41^ It is also important to understand whether illicit drug use and lack of analgesic access are relevant for HIV pain management in other contexts, such as those with universal health care. Therefore, we conducted a qualitative interview study to more deeply understand the experience and impact of neuropathic pain in people with HIV in the United Kingdom from a person-centred perspective.

## 2. Methods

This was a qualitative study. All participants provided written informed consent. This study was approved by the National Research Ethics Service (16/YH/0367) and Chelsea and Westminster NHS Foundation Trust R&D (C&W16/093). The study ran from December 2016 to July 2017.

### 2.1. Eligibility screening

Participants were eligible if they were 18 years or older, living with HIV, and screened positive for peripheral sensory neuropathy (self-reported bilateral foot pain in a symmetrical distribution).^76^ Participants were also required to screen positive for neuropathic pain in the feet, as indicated by scoring ≥3 on the patient-reported outcomes of the Douleur Neuropathique 4 Questions Interview (DN4i, English version).^4,5^ Our screening items enabled identification of “possible” neuropathic pain.^22^ We chose a positive symptom screen rather than formal diagnosis to determine eligibility as the study was concerned with the experience and impact of neuropathic pain symptoms. Finally, participants were eligible if pain was present most days for at least 3 months, with an average intensity and interference of ≥4 on a scale from 0 (no pain/no interference) to 10 (pain as bad as you can imagine/unable to carry on any activities).^71,77^

Consistent with previous studies, participants were excluded if they had a history of neuropathy due to a cause other than HIV or antiretroviral therapy, such as due to excessive alcohol consumption (>30 units/week^10^), hypothyroidism, vitamin B12 deficiency, diabetes, isoniazid treatment, or chemotherapy.^10,59^ These exclusion criteria were judged to be appropriate for this qualitative study to limit potential heterogeneity in the data due to other medical comorbidities that were not the focus of the study. We excluded participants if they had a diagnosis of dementia or a learning disability judged to interfere with their capacity to make informed consent or complete the interview. This study did not have the resources to conduct interviews or data analysis in languages other than English. Therefore, participants were excluded if they were unable to conduct the interview in English.

### 2.2. Recruitment

Men and women, people from diverse racial/ethnic backgrounds, and people who use recreational drugs were purposively sampled. Participants were recruited through HIV clinics at a major London hospital through clinician referrals and poster advertisements. We also recruited participants with HIV-related painful distal symmetrical polyneuropathy from a previous study by our group (HIV-POGO Study; https://clinicaltrials.gov/ct2/show/NCT02555930) who consented to be recontacted. The study was advertised at diverse community HIV organizations across London, United Kingdom. Finally, participants were also recruited through social media and chain referral sampling.^44^ Participants received a £20 gift voucher and travel expenses. We aimed to recruit approximately 30 participants, which was estimated on the basis of convention in qualitative methods and the range of demographic characteristics sought.^23,26^ Recruitment was terminated once interviews did not yield meaningfully novel information.^26^

## 3. Procedure

Participants completed a brief package of self-report questionnaires to bring to the interview appointment. Questionnaires were used for descriptive purposes only to characterise the sample. Questionnaires were completed before the interview appointment to minimise participant burden and fatigue on the interview day.

Participants reported on their age, gender, ethnicity, employment, living status, HIV and pain duration, antiretroviral therapy, CD4 count and viral load, pain medications, and current alcohol and illicit drug use. They completed the patient-reported outcomes of the DN4i to assess the presence of neuropathic pain symptoms, including burning, painful cold, and electric shocks.^4,5^ Participants also completed the Brief Pain Inventory (Cronbach's α = 0.94), a validated measure of pain intensity and interference in neuropathic pain.^11,78^ The 4 pain intensity and 7 interference items (rated from 0 to 10) were each averaged to produce 2 composite scores.^11^ Finally, they completed the Patient Health Questionnaire (Cronbach's α = 0.89), which is a validated 9-item measure of depression symptoms in chronic pain (total score ranges from 0 to 27).^30^

### 3.1. Interview appointment

#### 3.1.1. Clinical HIV-Associated Neuropathy Tool

At the start of the appointment, the interviewer administered the Clinical HIV-Associated Neuropathy Tool (CHANT).^47^ This was performed for descriptive purposes to assess the likelihood of participants having neuropathy; a positive CHANT screen in combination with the positive DN4i screen indicates “probable” neuropathic pain^22^; however, participants were not included/excluded based on their CHANT score. This 4-item tool assesses 2 subjective symptoms (self-reported bilateral foot pain and numbness) and 2 objective signs (loss of vibration sense and ankle reflex), which do not require specialist medical training. Patients are considered to have peripheral neuropathy if they are positive on a combination of 1 symptom and 1 sign in a bilateral distribution.^76^ The CHANT has 100% sensitivity and 85% specificity for diagnosing HIV-related peripheral neuropathy in relation to a comprehensive neurological examination.^76^ Participants were informed that the CHANT was administered to gather more information about the nature of their pain symptoms.

#### 3.1.2. Semistructured interview

The interview schedule was developed based on previous qualitative studies on psychological factors and treatments for pain in general and in HIV^40,62^ and input from community partners living with HIV and pain. In the first part of the interview, participants were asked to discuss the impact of pain on their lives, including on their mood and activities, and to describe their pain management strategies and the usefulness of these. The second half explored participants' views about an online treatment based on principles of Acceptance and Commitment Therapy for pain management (ACT; a recent development in CBT^38^) and solicited feedback on the acceptability of this treatment. Themes relating to the acceptability of online ACT for pain will be presented in another article (in preparation). Questions from the first part of the interview are provided in Appendix 1 (available at http://links.lww.com/PAIN/A931). The interview scheduled allowed for flexible probing of themes as they arose.

All interviews were individual and conducted face-to-face by the first author (W.S.), a registered clinical psychologist with expertise in chronic pain treatment and research. Participants were aware of the professional background of the interviewer. Field notes were made after each interview to record the interviewer's impressions of the interview process. Interviews were audio recorded and transcribed verbatim.

### 3.2. Data analysis

Thematic analysis was conducted using NVivo 12.0 (QSR International, London, United Kingdom) following the recommendations of Braun and Clark.^6^ An inductive approach to coding was used, and therefore, codes were added as they were identified in the data and there was no a priori set of codes. Each transcript was read several times to establish familiarity. First-level descriptive codes were recorded line-by-line for each transcript, followed by second-level codes reflecting subthemes. Third-level codes were used to identify higher order themes that combined subthemes. Quotations were extracted to illustrate subthemes.^6^ Both positive and negative examples of (sub)themes were coded for and integrated into the analysis during this process. Validity was established through the constant comparative method whereby transcripts were repeatedly reviewed to ensure (sub)themes corresponded to the data. The first and second authors each independently undertook this process for all transcripts and compared, discussed, and refined second- and third-level (sub)themes. The analysis was conducted iteratively with ongoing data collection and transcription. Recruitment ended when no new themes were identified from the analysed interviews within a diverse sample (ie, saturation).^53^ For this article, both parts of the interview were coded using this process. However, given the richness of the data, themes pertaining to the acceptability of online ACT for pain management will be presented in another article to ensure sufficient interpretation.

## 4. Results

A balance of genders and representation of people from diverse racial/ethnic backgrounds were achieved through purposive sampling. The sample included 14 men (54%) and 12 women (46%), with a mean age of 53.7 years (SD = 9.7; range = 40-78). Seventeen (65%) participants were white British/European, and 9 (35%) were black British/African. Participants had HIV (median = 17 years; range: 3-32 years) and self-reported neuropathic pain (median = 5 years; range: 1-24 years) of longstanding duration. Twenty-four participants (92%) were currently prescribed antiretroviral therapy, and the majority (88%) self-reported effective viral suppression. The sample had acceptable immune functioning as reflected by average CD4^+^ cell counts (mean = 575.0; SD = 189.2). The majority were unemployed (61.5%) and living alone (73.1%). Although people who use recreational drugs were purposively sampled, only 3 (12%) participants reported past or current recreational drug use. Fifteen (58%) participants reported that they currently drink alcohol. Scores on self-report questionnaires indicate that the sample was, on average, experiencing moderate levels of pain intensity and interference and depression symptoms.^30,77^ Table 1 further summarises sample characteristics with respect to pain and mood symptoms.

**Table 1:**
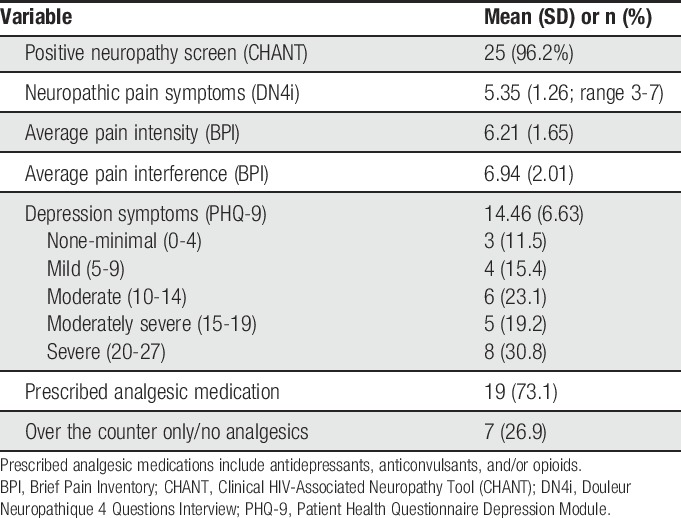
Descriptive statistics for pain and mood.

Twenty-five of 26 participants had a positive screen on the CHANT, indicative of peripheral neuropathy. The 1 participant who did not screen positive on the CHANT did screen positive on the DN4i during the initial eligibility screening. Therefore, this person was still considered eligible, as we did not use the CHANT to exclude people.

**Figure 1.**
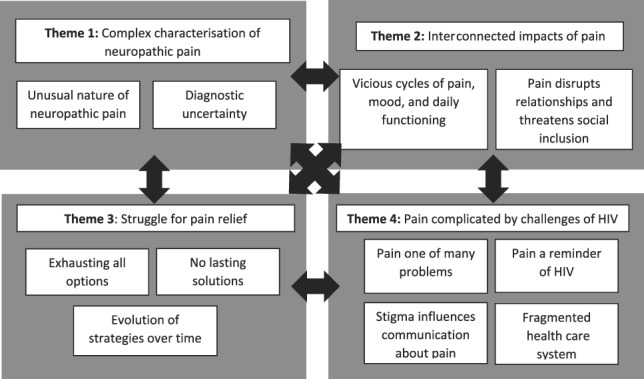
Summary of themes, subthemes, and their inter-relationships.

The interviews ranged from 37 to 80 minutes. We identified 4 themes and 11 subthemes (outlined in Fig. 1). Although we present the themes and subthemes separately, they are, by nature, strongly inter-related.

### 4.1. Theme 1: complex characterisation of neuropathic pain

This theme reflects participants' subjective descriptions of neuropathic pain and their understanding of its cause.

#### 4.1.1. The unusual nature of neuropathic pain

Neuropathic pain was reported as being experienced as unusual and distinct from other types of acute and chronic pain. Some expressed a sense of their feet as foreign or disconnected from their body. Participants spontaneously used vivid images to communicate this, although some had difficulty describing and quantifying these unusual sensations. A number of participants described other types of pain they experienced, including headaches and muscle pain. Some viewed these other pains as “normal” and related to age, for example. Participants varied in the degree to which they viewed neuropathic pain or other pains as the most problematic.

“I used to play football and…after playing football…you find the muscles are sort of achy so those are, I think, normal [pains]. But this is a different kind of pain. The other one you do feel it, fresh pain, there is a difference between fresh pain and something which looks stale. [Neuropathy pain] is a stale kind of pain which is on and off and you don't even know where it is coming from…sometimes it is just like heavy…quite honestly, I have been finding it difficult…sometimes there is no sensation… sometimes you feel it so wacky…sometimes it is hot…so there is no single way of defining the pain I have…it is [a] complex kind of thing.” (P21)

“Seems like someone is hitting you with a brush with a thousand steel prickles on it.” (P23)

“I'm really disconnected from them, my feet are not connected, they're like pigs trotters.” (P24)

#### 4.1.2. Diagnostic uncertainty

Some participants confidently attributed neuropathic pain to either HIV or specific ART regimens based on their remembered timeline of events. By contrast, others described confusion and uncertainty about the cause of pain. The lack of clear explanation was reported to be unhelpful and emotionally challenging in participants' attempts to understand and manage pain. One participant described a particularly distressing and ongoing path to diagnosis:

“…it's been a rollercoaster of why, what's going on, why has this happened […]. I'm trying to find […] answers all the time cos mentally […] this is draining me.” (P24)

### 4.2. Theme 2: interconnected impacts of pain

This theme reflects strong inter-relationships between the impacts of pain on mood, daily activities, and social functioning and inclusion.

#### 4.2.1. Vicious cycles of pain, mood, and functioning

Participants experienced a range of challenging emotions in response to pain and related functional limitations, including sadness, anxiety, anger, frustration, guilt, and disgust. There was a sense of vicious cycles between pain, mood, and functioning, such that pain and functional limitations impact on mood, and low mood exacerbates pain and limitations.

“…depression, anxiety, and pain are like the three witches. They are there, wound up together, because they relate, naturally. And those [depression and anxiety] feed into pain.” (P3)

Reference to positive emotions was infrequent. However, for example, a few participants spontaneously described feelings of enjoyment or pride. These emotions were linked with participation in personally meaningful activities (eg, participants 3, 11, and 17).

Participants described wide-ranging impacts of neuropathic pain on their daily functioning, from basic activities like wearing certain clothing to broader patterns of behaviour, such as engaging in employment-related activities. Participants varied with respect to whether pain or HIV was the predominant threat to their quality of life.

“HIV has **no** effect on my life […] neuropathy on the other hand…HIV has **zero** effect to me day to day. I take my pills and that's it. Umm yeah, the neuropathy, umm…is…it's not just pain, it's about function as well, day to day stuff, if you haven't slept very well, then you are not going to perform very well.” (P22)

It was sometimes difficult to determine the relative impact of neuropathic pain versus other pains on functioning. Some participants discussed the particular impact of peripheral neuropathy given the “centrality” of the feet to basic functions, such as walking. Many described significant impairments in walking and expressed fears about falling. Participants also frequently described the impact of neuropathic pain on their sleep. Some described vicious cycles of pain, worries, or frustration about walking or sleeping, which further worsened functional limitations.

“It's j-just…so irritating. [The peripheral neuropathy] doesn't seem to go away. I-i-it affects my **walking**…I walk very…**carefully** […] and I would be really slow because I'd be afraid of falling. […] I mean, if it's particularly bad on some days […] I've actually left the flat and I'm so self-conscious that my feet are not stable, I've just come home…I've not carried on with what I've wanted to do.” (P14)

#### 4.2.2. Pain disrupts relationships and threatens social inclusion

Many participants described how pain disrupts their relationships. This was experienced both in close relationships and in more distant ones. Participants reflected how pain made them more irritable, “snappy,” and aggressive in response to even minor social frustrations or stressors. Participants withdrew from social situations as a result.

“When my mood is low I stay at home and I'll stay away from people. I'm not going to interact with people because it can make you feel, they'll make you…s-s-s-snap, because you are feeling low, because you are in pain […] whereas normally I wouldn't be frustrated with people.” (P2)

There was consensus among participants that others do not understand their pain either because others do not have pain or do not believe the extent of pain and its associated limitations. There was concern not to burden others by discussing pain.

“People have sort of busy lives, you know. They've got so many problems of their own…they really don't take you too serious.” (P14)

A few participants described pain that threatened their sense of social inclusion due to not fulfilling perceived or idealised norms based on sexual orientation, gender, and age:

“In the gay world especially with 30 year olds, you are old, you are ancient. So if you start moaning about your pains, you know? You are a mommy or something.” (P9)

“The pain in my situation the way my body is and there because I'm a **woman** and I'm supposed to be, to feel, uhm…to feel…like enjoy life you know?…enjoy life, like look after the kids, look after the family and take care the husband, you know. But there are things I can't do and I feel I'm useless you know?” (P13)

### 4.3. Theme 3: struggle for pain relief

This theme reflects efforts participants engage in to obtain pain relief and the perceived usefulness of these efforts.

#### 4.3.1. Exhausting all options

Participants described their motivation to try all options for pain relief. There was a sense that even partial or temporary relief was worthwhile. At the same time, some questioned the helpfulness of continuing to pursue short-term pain relief. Pain relief strategies were highly individual and varied across participants. These strategies included trying all available medical treatments; complementary and alternative therapies; doing or, conversely, avoiding exercise; putting feet in ice water; psychological strategies learned through therapy or informally; peer support; and turning to religion or spirituality.

“I did do acupuncture […] uhm it does help but as, as, as I say it just is such a short time of relief of pain which is [long pause] of course it's worth it, anything to try to take the pain away, is, is fine but uhm the relief of pain is such a short time which sometime [sic] I think [sighs]…I really can't bother.” (P12)

#### 4.3.2. No lasting solutions

No participant had found a simple solution for sustained pain relief. Many described frustration that most pain control strategies only provide minimal relief or that meaningful relief was not maintained long term. There was significant concern about side-effects and long-term use of analgesic medications, particularly in the context of taking daily ART with its own side effects. As a result, many expressed a strong desire to not take more medication.

“I've been treated very different ways but it seems to be working for a couple of month [sic] and after that everything is coming back again, again the same…the same story.” (P12)

“I don't take them [pain medication] regularly, I-I take them when the pain is really really bad, uhm… I don't, I mean the pain medication doesn't really help that much, it-it, I think what it does it just makes you feel a little bit out of it, and then you are not focusing on it so much but, but being a bit out of it is not really ideal [laughs].” (P15)

#### 4.3.3. Evolution of strategies over time

Some participants described that their pain management strategies evolved over time, often through an exhausting process of trial and error. Through this process, some felt that they had found more helpful strategies. For a few participants, more helpful strategies included stopping pain control strategies experienced as harmful (eg, escalating analgesic medications) and/or focusing on their life more broadly, such as doing activities they enjoy. However, a number of participants did not feel that they had found helpful strategies for managing pain or its impact. Many were still in the process of wanting to “try everything,” with some even returning to strategies previously identified as problematic (eg, taking opioids).

“…having been through the gamut of them [analgesic medications] I…also realise that although the fentanyl patches were working, unsurprisingly because it's morphine, that they were working **less**, and I would therefore be increasing my intake of morphine…I stopped using them because I don't want to go down that sort of a slippery slope.” (P3).

“For many years…I…must have become a victim to [the neuropathy], so I didn't go out for walks, umm, which meant I didn't join into…join in things with people as much and family and so on…but [now] I started going for walks which I think helped. [Walking] helped me mentally as well as it's really beautiful where I live.” (P17)

### 4.4. Theme 4: pain is complicated by challenges of HIV

The impact and management of pain is intimately connected to broader challenges of living with HIV. Each of the other themes cannot be understood in isolation from this context.

#### 4.4.1. Pain is one of many problems

In addition to pain, participants enumerated many symptoms they struggled with in connection with HIV and ART, including fatigue, gastrointestinal problems, lipodystrophy, tinnitus, and memory and concentration difficulties. Participants expressed a sense of problems piling up, which was exacerbated by the challenges of aging, exemplified by one participant:

“…who is helping people over 60? We have been with this since ‘82…I am **sick to death** of hearing it is a f***ing disease that everyone can live a long life, it hasn't been for me, it has been a f***ing nightmare.” (P6)

#### 4.4.2. Pain is a reminder of HIV

Participants varied in their willingness to openly discuss their HIV diagnosis, which some wished to forget. For some, pain served as a distressing reminder of HIV, the need to take ART, one's own mortality, and/or the loss of loved ones to HIV. These issues were accompanied by grief, frustration, and anger. Some felt their experience taking ART and its side effects were at odds with the portrayal of ART as “wonder drugs” within the medical community. Consequently, some wished to temporarily stop ART, but felt they had no control to do so.

“You are being invaded by a virus and a virus is doing this to your body when you…don't really want to be dealing with this amount of pain and to-to manage all the things you have to take medication, I don't know what is caused by side effects of the medication or the HIV […] I have asked my consultant many times if I could have a break and see how I would feel without-without the medication, even just for a while, just to see whether there is a degree of toxicity, uhm but…I was very quickly talked out of it.” (P15)

#### 4.4.3. Stigma influences communication about pain

HIV stigma was an important consideration limiting the communication of pain to others. Participants tended to express pain only to a close circle of people who knew of their HIV status. Among some participants, there was a sense of belonging, acceptance, and safety with their HIV positive peers, from whom they could seek support in managing pain and other challenges.

“…like most people with HIV, there's people who do know about HIV and people who don't, and the lot who don't know, you have to think on your feet because you might talk about pain. I mean, who goes to doctors three or four times a week…we do. So you have to be careful…I could talk about my high blood pressure but I don't talk about [HIV], so it's, it's really hard to keep those things separate. When you're amongst your peers its okay.” (P26)

#### 4.4.4. Navigating a fragmented health care system

Participants felt they are constantly attending appointments and that their care is not unified. In some cases, confusion was reported as to which doctors had been seen for which purposes and who was managing various problems. These challenges have been exacerbated by funding cuts and uncertainty about the availability of treatments perceived as helpful for pain or mental health, including complementary and alternative therapies and psychotherapy.

“I was taking [tramadol] for 12 years. They never checked again whether I can still continue, whether they are okay, so with my GP at the moment, I-I-I didn't find enough support, with this [sic] pain problems, every time I used to come to [the hospital] uh, HIV clinic doctor and then he would always refer me to pain doctor.” (P25)

## 5. Discussion

This is the first qualitative study to explore the experience and impact of neuropathic pain in people with HIV. A strength of the study is its diverse sample, comprising participants with varied racial/ethnic backgrounds, near gender balance, and range of ages and HIV duration. Four themes were identified reflecting the complex characterisation of neuropathic pain, interconnected impacts of pain, the struggle for pain relief, and the complexity of managing pain in the context of HIV. These data can inform holistic approaches to pain management in HIV and other stigmatised infectious diseases where there is a high burden of neuropathic pain.^27^

The identified (sub)themes were strongly inter-related. For example, the perceived “unusual” nature of neuropathic pain was compounded by lack of diagnostic clarity (theme 1), leading to a continual search for an explanation and treatment, exacerbating distress (themes 2 and 3). This response to diagnostic uncertainty is described elsewhere in the chronic pain literature.^49^ People with HIV may be wary of disclosing “unusual” neuropathic symptoms for fear of further stigma (theme 4). Lack of routine inclusion of interdisciplinary pain management in HIV services may exacerbate these challenges (theme 4). Thus, understanding the wider HIV context is crucial to make sense of how people experience and manage pain. Previous qualitative studies in the United States, Kenya, and Uganda also highlight the need to consider the HIV context to understand pain management barriers.^40,57^

Participants in this study frequently attributed pain to HIV or ART. By contrast, participants in the study by Merlin et al.^40^ largely did not view HIV or ART as the cause of their chronic pain. The focus on neuropathic pain in the current study may account for this difference. The link between neuropathic pain and HIV was emotive and difficult to discuss for some, signifying past hardships related to HIV and an uncertain future ageing with the virus. Therefore, the meaning attributed to the specific type of pain is important when investigating the impact of pain in HIV.

Participants reported broad ranging and synergistic impacts of pain on their mood and functioning, consistent with the wider literature.^16,20,65^ The impact of neuropathic pain on sleep and walking and the associated anxiety and frustration was striking. Descriptions of fear of falling and reduced walking are consistent with the fear-avoidance model largely applied to musculoskeletal pain.^70^ One study in diabetic neuropathy found fear of falling was associated with increased disability, controlling for medical factors, pain intensity, and pain-related fears.^24^ Although numbness and balance issues related to peripheral neuropathy can themselves increase risk of falls, fear of falling can further exacerbate the risk.^7,31^

The qualitative study by Merlin et al. also showed that participants with HIV and chronic pain understand bidirectional relationships between pain and low mood.^40^ The current results extend this by showing how the impact of pain on functioning and mood threatens social inclusion, particularly when it prevents adherence to norms for gender and sexual orientation. Greater understanding of the intersections between pain, gender, ethnicity, and age is needed given individual and systemic barriers that may contribute to pain management disparities regarding these factors.^1,2,8,39,54^ Relatedly, data support the cross-cultural validity of common neuropathic pain descriptors.^13,58^ However, further research should explore in-depth cultural differences in the images and metaphors used to describe neuropathic pain and how this influences pain management.

Our sample varied in the success of efforts to manage pain, although a constant struggle to find more helpful pain management was common. Participants described poor long-term efficacy of pain medications, consistent with numerous randomised-controlled trials.^21,46^ Taking pain medications on top of daily ART may feel especially burdensome,^32^ and pain management is complicated by interactions between ART and analgesics.^37^ Survivors of the pre-cART era and their clinicians face a shift from an end of life palliative care model towards one that focuses on improving functioning with chronic pain.^45^ This suggests a need to increase the efficacy and accessibility of nonpharmacological approaches to chronic pain management in this population.

Participants used a wide range of pain management strategies, including medical, complementary and alternative approaches, psychological techniques, and social support. Reports of illicit substance use were infrequent. This is despite using a range of recruitment methods to sample people who use illicit drugs. The lack of representation of this group in the sample is an important finding itself. This differs from Merlin et al.'s findings in a sample of predominantly African American men with HIV and pain where self-medication with illicit drugs was a theme.^40^ With the US opioid crisis, there are significant challenges accessing appropriate pain management, particularly among racial/ethnic minorities.^9,39,61^ This may lead to increased illicit substance use in that context.^50^ Further investigation of the interaction between individual and systemic factors related to pain management is needed in lower- and middle-income countries which face unique challenges.^42,57^

Experiences of stigma were reflected in the content of responses and the unwillingness of some participants to explicitly talk about HIV during the interviews. Addressing stigma has been pivotal in the HIV/AIDS response.^35^ Despite this, there is limited quantitative research examining pain and stigma in HIV.^55^ People with HIV may continue to function with pain to conceal their HIV status, although this may lead to greater distress.^73,74^ Research on stigma in pain is in its infancy, but there are indications that stigma is associated with poorer pain outcomes.^14,56^ Further research should investigate the combined effects of HIV- and pain-related stigma on quality of life.

The current data suggest that evidence-based cognitive-behavioural approaches^75^ for chronic pain in the general population are also relevant for people with HIV. The data can inform the tailoring of cognitive-behavioural approaches to meet the needs of people with HIV and neuropathic pain. Sleep impairment and walking-related anxiety may be particularly important to target in pain management for this population. Graded exposure (for walking)^3^ and sleep hygiene/restriction (for insomnia)^69^ may be bolstered by strategies from ACT, such as present-moment awareness and values-based living, which may increase willingness to engage in behaviour changes that initially include aversive experiences.^28^ ACT helps people identify pain management strategies that are ineffective or costly with respect to their valued goals, and shifts focus toward engaging in meaningful activities alongside the challenges and uncertainties of living with pain.^38,68^ Compassion-focused approaches may help manage the impact of HIV and pain stigma, which may be enhanced by interventions targeting systemic stigma.^33,60^ Even in the absence of a clear diagnosis, validation of the severity and impact of neuropathic pain by HIV clinicians may mitigate the distress caused by these “unusual” symptoms.^17,34^ Integration of holistic, interdisciplinary pain management within HIV services may reduce the challenges and confusion experienced with accessing disparate services.

This study has several limitations. Responses may have been influenced by participants' knowledge of the interviewer's professional background. In addition, the sample had moderate pain intensity and interference and depression symptoms, on average. This represents a group for whom treatments can be meaningfully targeted to improve pain-related quality of life. However, this may have led to an overrepresentation of (sub)themes reflecting poor pain management. Future research on people with HIV who are successfully functioning with pain may provide important insights into adaptive processes to foster through treatment.^25^

We excluded non-English speakers as we did not have resources for interpreters across the possible languages. No participant was excluded during screening because of speaking insufficient English to conduct the interview. However, non-English speakers may not have been referred to the study thus limiting representation of an even more diverse group. We did not systematically collect data on treatment within specialty pain management services. Therefore, it is unclear the extent to which (lack of) engagement with pain management services may have influenced the results. Our study was conducted in England, where participants have access to universal health care. Future research is needed to understand the impact of HIV-related pain in countries with different health care systems.

To conclude, this study supports the relevance of targeting psychosocial factors to manage the impact of neuropathic pain in HIV. Further development and testing are needed to determine the feasibility of psychosocial approaches to manage neuropathic pain and associated symptoms experienced by people with HIV. Research is needed to determine how best to tailor such treatments to different subgroups of people affected by HIV and chronic pain.

## Conflict of interest statement

A.S.C. Rice undertakes consultancy and advisory board work for Imperial College Consultants. In the past 24 months, this has included remunerated work for: Pharmanovo, Galapagos, Toray, Quartet, Lateral, Novartis, Pharmaleads, Mundipharma, Orion, Asahi Kasei, and Theranexis, outside the submitted work. A.S.C. Rice was the owner of share options in Spinifex Pharmaceuticals from which personal benefit accrued upon the acquisition of Spinifex by Novartis in July 2015. In addition, Professor Rice has the following patents pending: Rice ASC, Vandevoorde S, and Lambert DM Methods using N-(2-propenyl)hexadecanamide and related amides to relieve pain WO 2005/079771, and Okuse et al. Methods of treating pain by inhibition of vgf activity EP13702262.0/WO2013 110945. The remaining authors have no conflicts of interest to declare.

## Appendix A. Supplemental digital content

Supplemental digital content associated with this article can be found online at http://links.lww.com/PAIN/A931.

## Supplementary Material

SUPPLEMENTARY MATERIAL
