# Supplementary material for: A qualitative study of the experience and impact of neuropathic pain in people living with HIV
Source: Pain. 2020 Dec 20;161(5):970–8. doi: 10.1097/j.pain.0000000000001783 (PMC7170440; doi:10.1097/j.pain.0000000000001783)
Supplement: SUPPLEMENTARY MATERIAL [file jop-161-0970-s001.docx]

**Appendix A: Interview Schedule**

Part 1: Impact of pain

1) How does the pain affect your life?

Subprompts:

-How does pain affect your activities?

-How does pain affect your mood?

2) What do you currently do to manage your pain?

Subprompts:

-If taking analgesic medication: How well do medications work for you? Do you get any side effects?

-Do you do anything else to manage your pain?

-Do these things help? Do they have any consequences or downsides?
